# Novel Estimation of Penumbra Zone Based on Infarct Growth Using Machine Learning Techniques in Acute Ischemic Stroke

**DOI:** 10.3390/jcm9061977

**Published:** 2020-06-24

**Authors:** Yoon-Chul Kim, Hyung Jun Kim, Jong-Won Chung, In Gyeong Kim, Min Jung Seong, Keon Ha Kim, Pyoung Jeon, Hyo Suk Nam, Woo-Keun Seo, Gyeong-Moon Kim, Oh Young Bang

**Affiliations:** 1 Clinical Research Institute, Samsung Medical Center, School of Medicine, Sungkyunkwan University, Seoul 06351, Korea; yoonckim1@gmail.com; 2 Department of Neurology, Samsung Medical Center, School of Medicine, Sungkyunkwan University, Seoul 06351, Korea; khhhj7@naver.com (H.J.K.); neurocjw@gmail.com (J.-W.C.); smcingyeong@naver.com (I.G.K.); mcastenosis@gmail.com (W.-K.S.); kimgm@skku.edu (G.-M.K.); 3 Department of Radiology, Samsung Medical Center, School of Medicine, Sungkyunkwan University, Seoul 06351, Korea; m.seong@samsung.com (M.J.S.); somatom.kim@samsung.com (K.H.K.); pyoung.jeon@samsung.com (P.J.); 4 Department of Neurology, Yonsei University, Seoul 03722, Korea; hsnam@yuhs.ac

**Keywords:** stroke, ischemia, machine learning, cerebral infarction

## Abstract

While the penumbra zone is traditionally assessed based on perfusion–diffusion mismatch, it can be assessed based on machine learning (ML) prediction of infarct growth. The purpose of this work was to develop and validate an ML method for the prediction of infarct growth distribution and volume, in cases of successful (SR) and unsuccessful recanalization (UR). Pre-treatment perfusion-weighted, diffusion-weighted imaging (DWI) data, and final infarct lesions annotated from day-7 DWI from patients with middle cerebral artery occlusion were utilized to develop and validate two ML models for prediction of tissue fate. SR and UR models were developed from data in patients with modified treatment in cerebral infarction (mTICI) scores of 2b–3 and 0–2a, respectively. When compared to manual infarct annotation, ML-based infarct volume predictions resulted in an intraclass correlation coefficient (ICC) of 0.73 (95% CI = 0.31–0.91, *p* < 0.01) for UR, and an ICC of 0.87 (95% CI = 0.73–0.94, *p* < 0.001) for SR. Favorable outcomes for mismatch presence and absence in SR were 50% and 36%, respectively, while they were 61%, 56%, and 25%, respectively, for the low, intermediate, and high infarct growth groups. The presented method can offer novel and alternative insights into selecting patients for recanalization therapy and predicting functional outcome.

## 1. Introduction

Measurement of ischemic core and penumbra volumes in acute ischemic stroke provides clinicians with important clues for predicting clinical response after successful recanalization (SR) and selecting patients for treatments, such as intravenous thrombolysis and endovascular therapy (EVT) [1,2,3]. Diffusion-weighted imaging (DWI) and perfusion-weighted imaging (PWI) in magnetic resonance imaging (MRI) are neuroimaging modalities that can visualize the location and extent of ischemic stroke lesions with high sensitivity and specificity. The target mismatch criteria help determine patient selection for EVT and are evaluated using DWI and PWI lesion volumes [2]. 

Besides measurement of the PWI–DWI mismatch zone, estimation of infarct growth would provide a direct approach to the prediction of penumbra areas and the final infarct lesion volume [4]. There have been conflicting results on whether mismatch volume predicts infarct growth volume. It was reported that the infarct growth volume was greater than the mismatch volume, and half of the patients without target mismatch suffered from infarct growth regardless of the revascularization [5,6], while a recent randomized trial of EVT showed a good correlation between the predicted infarct volume and the 27-h infarct volume in target mismatch patients [7]. A clinical trial with a serial MRI study reported that ischemic lesion volume increased, by various degrees, from baseline to 12 weeks in untreated patients [8].

Machine learning (ML) has emerged as a promising methodology in acute stroke neuroimaging to predict ischemic stroke growth by interpolation of shape representations [9], and to predict the voxel-based tissue outcome [10,11] and the clinical outcome [12]. In particular, an ML method, referred to as fully automated stroke tissue estimation using random forest classifiers (FASTER) [13], showed potential to accurately predict final lesion volumes in cases of SR and unsuccessful recanalization (UR). However, the study did not investigate the performance of ML-based infarct growth estimation in comparison with target mismatch based on PWI–DWI mismatch volume and did not evaluate the prediction of clinical outcomes according to ML-based infarct growth estimation.

In the present study, we investigated the feasibility of an ML-based tissue outcome prediction technique using features derived from DWI and PWI data. Based on the ML method, we evaluated the performance of predicting final infarct volumes and infarct growth in the SR and UR cases. We also investigated how well the selection based on the ML-predicted infarct growth volume corresponded with the target mismatch classification in actual infarct growth and functional outcome distributions.

## 2. Materials and Methods

### 2.1. Patients

This study included patients who were admitted to a university medical center between 5 June 2005 and 31 December 2016. The study inclusion criteria were as follows: (1) symptomatic middle cerebral artery (MCA) occlusion, (2) MRI scan including DWI and PWI sequences prior to treatment and including DWI at 7 days after symptom onset, and (3) baseline MRI within 6 h of symptom onset.

Patients were divided into two groups: UR and SR. The UR group consisted of patients with modified treatment in cerebral infarction (mTICI) scores of 0–2a. The SR group consisted of patients with mTICI scores of 2b–3. For patients treated with EVT, the mTICI score was determined based on post procedural digital subtraction angiography (DSA), and for patients treated with intravenous (IV) tissue plasminogen activator (tPA), it was determined based on 24 h MRI and MR angiography (MRA).

The UR group was divided into the UR development and UR external validation groups, and the SR group was divided into the SR development and SR external validation groups. All patients in the SR group underwent EVT. The development/validation division criterion was based on the chronology of acute ischemic stroke occurrence: The UR and SR development groups corresponded to stroke onset dates prior to 31 December 2010 and 31 December 2011, respectively.

Demographic information including age and sex, baseline National Institutes of Health Stroke Scale (NIHSS) scores, and the 90-day modified Rankin Scale (mRS) scores were collected. The baseline NIHSS score is used to quantify the impairment due to a stroke and ranges from no symptoms at all (0) to the most severe stroke (42). The 90-day mRS score is used to assess clinical outcome and ranges from no symptoms at all (0) to death (6). Radiologic information, such as time interval from symptom onset to MRI, was obtained from electronic medical records.

All patients or patient guardians provided informed consent for inclusion before they participated in the study. The study was conducted in accordance with the Declaration of Helsinki, and the protocol was approved by the institutional review board of the Samsung Medical Center (IRB no. 2016-08-064).

### 2.2. Data Acquisition

MRI data were acquired on a 3T scanner system (Philips Achieva, Best, the Netherlands). DWI sequence parameters were as follows: repetition time = 3 s, echo time = 81–88 ms, number of slices = 22, image matrix size = 256 × 256, pixel spacing = 0.9375 mm × 0.9375 mm, and spacing between slices = 6.5 mm. The apparent diffusion coefficient (ADC) was calculated in a pixel-by-pixel manner from DWI acquired with b = 0 and b = 1000 s/mm^2^. The PWI sequence was based on the commonly used T2*-weighted dynamic susceptibility contrast with the injection of a bolus of extracellular gadolinium contrast agent, and the parameters were as follows: repetition time = 1.5–1.7 s, echo time = 35 ms, number of slices = 22, spacing between slices = 6.5 mm, echo train length = 67, number of frames = 50, image matrix = 256 × 256, pixel spacing = 0.9375 mm × 0.9375 mm, and spacing between slices = 6.5 mm.

### 2.3. ADC/rTTP and Data Preprocessing

**Figure A1:**
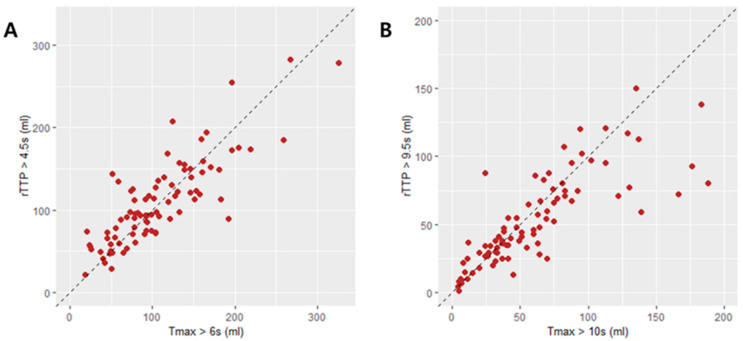
Comparison of time-to-maximum (Tmax) and relative time to peak (rTTP) lesion volumes. (**A**) Perfusion lesion volumes measured by Tmax and rTTP. (**B**) Severe ischemic lesion volumes measured by Tmax and rTTP.

Typically, ADC and time-to-maximum (Tmax) are used to determine diffusion and perfusion lesion volumes, respectively. A recent study suggested that relative time to peak (rTTP) > 4.5 s was empirically identical to Tmax (>6 s) in determining perfusion lesion volume and resulted in > 90% accuracy, when compared with the conventional Tmax (>6 s) [14]. Notably, in the present study we used the statistics of rTTP as features for ML training, as opposed to a previous study, which used the statistics of Tmax, cerebral blood flow (CBF), cerebral blood volume (CBV), and time to peak (TTP) values as features for ML training [13]. Tmax, CBF, and CBV calculations involve numerical deconvolution with arterial input function (AIF), which is known to be sensitive to image artifacts and the choice of region of interest in major cerebral arteries [15]. The present study only considered the calculation of rTTP (i.e., the TTP delay relative to the contralateral region) for the extraction of PWI-related imaging features, and it did not consider estimating the AIF and performing numerical deconvolution. We sought to evaluate the correspondence between the rTTP-derived PWI lesion volume and the Tmax-derived PWI lesion volume, and the rTTP > 4.5 s volume showed Pearson correlation coefficient of 0.84 with the Tmax > 6 s volume in the subjects considered in the study (Figure A1).

Baseline DWI/ADC, PWI, and day-7 DWI images were co-registered using Statistical Parametric Mapping (SPM) 12 [16]. The rTTP maps from baseline PWI data were automatically calculated as follows. At each voxel, the MR signal was converted to contrast agent concentration, and TTP was measured from the concentration curve. Median TTP values were computed from the right and left hemispheric regions, respectively. The hemisphere with the lower median TTP value was defined as the contralateral region, and the median TTP value in this region served as the baseline TTP. The rTTP maps were calculated by voxel-wise subtraction of the baseline TTP from TTP values.

The midsagittal plane in the axial brain slice was automatically estimated after determining the optimal values of the translation and rotation parameters. The parameter values were used to identify the midline of an image. The midline helped to identify the correct location of a symmetric contralateral voxel, given a stroke lesion voxel, when feature extraction was performed for ML model development.

### 2.4. ML Model Development

**Figure 1:**
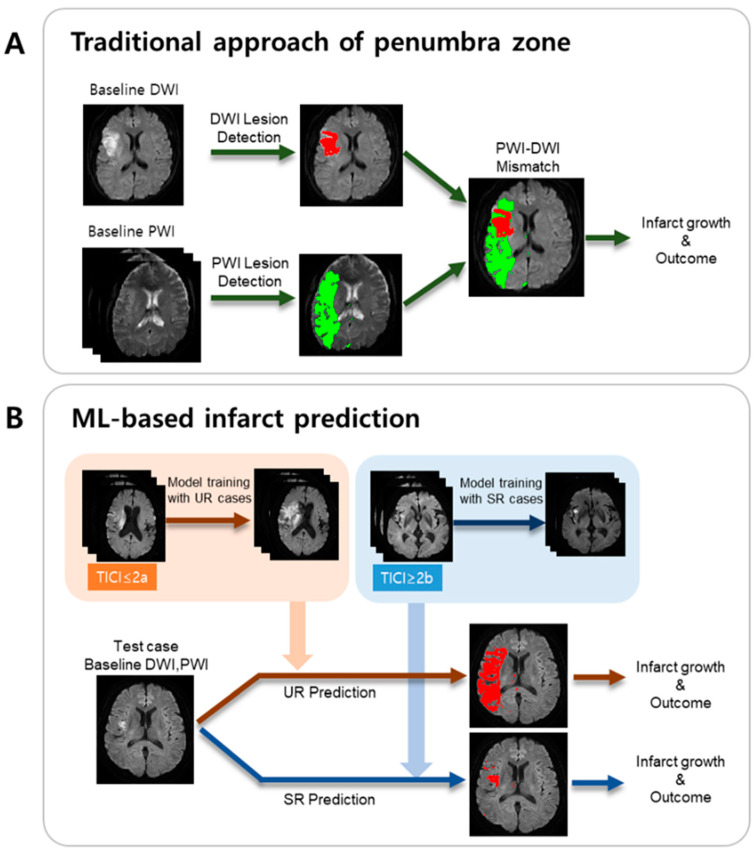
Schematics of two approaches for the estimation of final infarct volume and functional outcome. (**A**) Traditional approach of penumbra zone estimation. (**B**) Machine learning (ML)-based infarct prediction.

A schematic of the presented ML method is illustrated in Figure 1B, in comparison with the traditional approach based on the PWI/DWI target mismatch (Figure 1A).

Feature extraction was conducted as follows. Seven consecutive axial slices covering the brain tissue in the MCA territory were considered for feature extraction. All the voxels in the tissue of the lesion hemisphere were considered as candidates, where the infarct voxels were labeled as “1”, and the non-infarct voxels were labeled as “0”. For training, to avoid class imbalance issues, we set the number of infarct voxels to be equal to the number of non-infarct voxels. For each candidate voxel, we considered its neighborhood as 5 × 5 × 3 voxels surrounding the candidate voxel. From the ADC values of the neighborhood, a set of 12 features was computed, consisting of range, mean, median, min, max, standard deviation (SD), skew, kurtosis, 10th percentile, 25th percentile, 75th percentile, and 90th percentile. Another set of 12 features was computed from the rTTP values in the neighborhood. These 24 features were also computed from a neighborhood of the voxel in the contralateral hemisphere. Hence, the number of features was 24 × 2 = 48. For the UR model, the dimensions of (# of samples) × (# of features) in the training dataset were 297,192 × 48, which provided the input for the UR model. For the SR model, the dimensions in the training dataset were 193,510 × 48, which provided the input for the SR model. Binary infarct masks, manually delineated while referring to the day-7 DWI images, were obtained from all the datasets using the ITK-SNAP software [17]. The output class labels for supervised ML were obtained from the binary infarct masks.

With the training dataset, a five-fold cross-validation was performed to evaluate the performance of the UR and SR models. The splitting in the cross-validation folds was constructed on the basis of patients rather than voxels. The assignment of patients to each fold was performed randomly. The ML model of the random forest classifier, provided by the Python scikit-learn module, was used for cross-validation [18]. After hyperparameter selection of the model using a random search, the mean and SD of cross-validation accuracy was computed for each model.

### 2.5. External Validation

For external validation, we used baseline DWI and PWI images from the 12 UR and the 27 SR patients. Candidate voxels were chosen, and for each candidate voxel, the feature extraction procedures were performed in the same manner as those for the model development. For each voxel, the extracted features were input to the trained UR and SR models to result in a tissue outcome probability score *p*, where *p* ≥ 0.5 indicated an infarct voxel, and *p* < 0.5 indicated a non-infarct voxel. After processing all the candidate voxels, we obtained binary infarct prediction maps for both the UR and SR cases. The final infarct volume was calculated as the number of predicted infarct voxels multiplied by the voxel volume. Two final infarct volumes were calculated: one for the UR case and the other for the SR case.

For the UR (or SR) patient group, the final infarct volumes from the UR (or SR) model were compared with the final infarct volumes from the manually annotated day-7 DWI infarct masks. The Dice similarity coefficient (i.e., Dice score) was computed for each patient to evaluate the overlap between the ML predicted infarct mask and the manually segmented day-7 DWI infarct mask.

### 2.6. Mismatch and Infarct Growth Estimation

Target mismatch classification requires information on baseline diffusion and perfusion lesion volumes. We computed the Tmax > 6 s volume (i.e., perfusion lesion volume), the Tmax > 10 s volume (i.e., severe ischemic lesion volume), and the ischemic core volume based on the ADC < 600 × 10^−6^ mm^2^/s threshold. Target mismatch criteria were defined as (1) a perfusion lesion volume to ischemic core volume ratio of 1.8 or more, (2) a difference between perfusion lesion volume and ischemic core volume of 15 mL or more, (3) the ischemic core volume less than 70 mL, and (4) the Tmax > 10 s volume less than 100 mL.

Actual infarct growth volume was calculated as the difference between the day-7 final infarct volume and the baseline diffusion lesion volume (i.e., ischemic core volume). The day-7 final infarct volume was measured after manual segmentation of the infarct lesions. Predicted infarct growth volume was calculated as the difference between the SR (or UR) predicted infarct volume and the baseline diffusion lesion volume. The SR (or UR) patients were categorized into tertiles according to the predicted infarct growth volume.

The percentage of favorable functional outcome was evaluated for the patient groups with and without target mismatch and for the patient groups categorized into tertiles based on SR (or UR) predicted infarct growth. Favorable functional outcome was defined as a 90-day mRS score of ≤ 1.

### 2.7. Statistical Analysis

We conducted statistical analysis using the R software package (R Foundation for Statistical Computing, Vienna, Austria). Descriptive demographics, and clinical and radiological data are shown as mean ± SDs, numbers, or median and interquartile ranges. An unpaired two-sample Student’s t-test was performed to determine if the development and external validation data were significantly different in terms of age, NIHSS score, lesion volume, or onset to MRI time. A *p*-value < 0.05 was considered statistically significant. The intraclass correlation coefficient (ICC) and its 95% confidence interval (CI) were computed between the two infarct volume measurements. A *p*-value < 0.05 was considered to identify a statistically significant correlation, given the null hypothesis of no relationship between the two measurements. Bland–Altman analysis was performed by computing the mean difference and 95% limits of agreement (LOA) between the two volume measurements.

### 2.8. Data Statement

The datasets in the present study are not publicly available since they have private patient information. The de-identified data are available from the corresponding author upon request after approval of the institutional review board of Samsung Medical Center.

## 3. Results

A total of 102 patients satisfied the criteria of < 6 h stroke onset to MRI time, MCA occlusion, and baseline NIHSS score of ≥ 4. Of 102 patients, 40 patients had an mTICI score of 0–2a (comprising the UR group), and 62 patients had an mTICI score of 2b–3 (comprising the SR group). A total of 10 patients were excluded, since their image data were problematic in terms of (1) severe motion artifacts in PWI data (*n* = 8), (2) susceptibility artifact near frontal brain regions (*n* = 1), and (3) infarcts detected in unexpected locations on day-7 DWI, which were related to stroke recurrence or procedural complications (*n* = 1). Hence, out of 92 patients, the final numbers of SR and UR patients were 56 and 36, respectively (Table 1). In both the SR and UR groups, there were no statistically significant differences in age, male sex, baseline NIHSS score, onset to MRI time, baseline DWI and PWI lesion volumes, and day-7 DWI lesion volumes between the development and external validation groups, except for the baseline NIHSS score between the UR development and external validation groups (Table 1).

For the UR model, the five-fold cross-validation resulted in a mean accuracy (SD) of 74.6% (2.5%) and a 95% CI of 69.8%–79.4%. For the SR model, the five-fold cross-validation resulted in a mean accuracy (standard deviation) of 76.4% (6.5%) and a 95% CI of 63.7%–89.2%.

**Figure 2:**
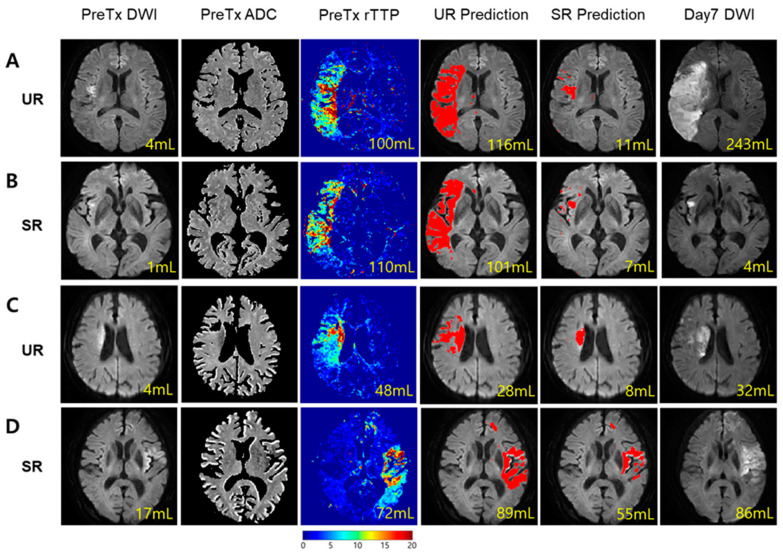
Sample external validation cases of SR- and UR-based infarct predictions. (**A**) A case of large infarct growth in a UR patient (intravenous thrombolysis, modified treatment in cerebral infarction (mTICI) = 0, and 90-day modified Rankin Scale (mRS) = 5). (**B**) A case of small infarct growth in an SR patient (endovascular therapy, mTICI = 2b, and 90-day mRS = 0). (**C**) A case of small infarct growth in a UR patient (endovascular therapy, mTICI = 1, and 90-day mRS = 2). (**D**) A case of large infarct growth in an SR patient (endovascular therapy, mTICI = 2b, and 90-day mRS = 5). Predicted infarct masks, shown in red, are overlaid on pre-treatment DWI images for UR and SR model predictions. All four patients had target mismatch presence but showed various degrees of infarct growth. The yellow numbers indicate the following volumes: (from left to right) baseline DWI lesion volume, baseline PWI lesion volume, UR-predicted infarct volume, SR-predicted infarct volume, and day-7 infarct volume.

Final infarct predictions using the UR and SR models are shown for four different cases (Figure 2; patient A: UR with large infarct growth; patient B: SR with small infarct growth; patient C: UR with small infarct growth; and patient D: SR with large infarct growth). The large differences between the UR-predicted and SR-predicted volumes were observed in both patients A and B, but the failure of revascularization led to the large final infarct volume (243 mL) and unfavorable outcome (90-day mRS = 5) in patient A, whereas the success of revascularization led to the small final infarct volume (4 mL) and favorable outcome (90-day mRS = 0) in patient B. Regarding the actual infarct growth, patient A’s (239 mL) was significantly larger than patient C’s (28 mL), while patient D’s (69 mL) was significantly larger than patient B’s (3 mL). All four cases had target mismatch and showed substantial differences in infarct growth volume, regardless of recanalization status.

**Figure 3:**
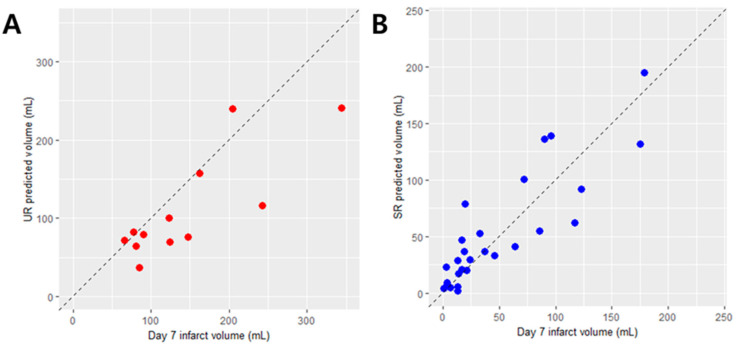
Correlation plots of (**A**) the UR-predicted infarct volume and (**B**) the SR-predicted infarct volume on the external validation UR and SR cohorts, respectively. The manual infarct volume measurement on day-7 DWI served as the reference.

In the UR model validation, the ICC between manual infarct volume and UR-predicted infarct volume was 0.73 (95% CI = 0.31–0.91, *p* < 0.01; Figure 3A). In the SR model validation, the ICC between manual infarct volume and SR-predicted infarct volume was 0.87 (95% CI = 0.73–0.94, *p* < 0.001; Figure 3B). The mean difference (95% LOA obtained from Bland–Altman analysis) was −32.5 mL (−126.9 mL, 61.9 mL) for the UR model validation and 3.5 mL (−48.2 mL, 55.2 mL) for the SR model validation.

In all the external validation subjects, the presented ML model had a median Dice similarity coefficient (DSC) of 0.49 (IQR, 0.37–0.59), which was comparable to the DSC of 0.53 (IQR, 0.31–0.68) in the study by Yu et al. [19]. The presented ML model had a median DSC of 0.43 (IQR, 0.20–0.52) in the SR external validation subjects and a median DSC of 0.58 (IQR, 0.55–0.67) in the UR external validation subjects.

**Figure 4:**
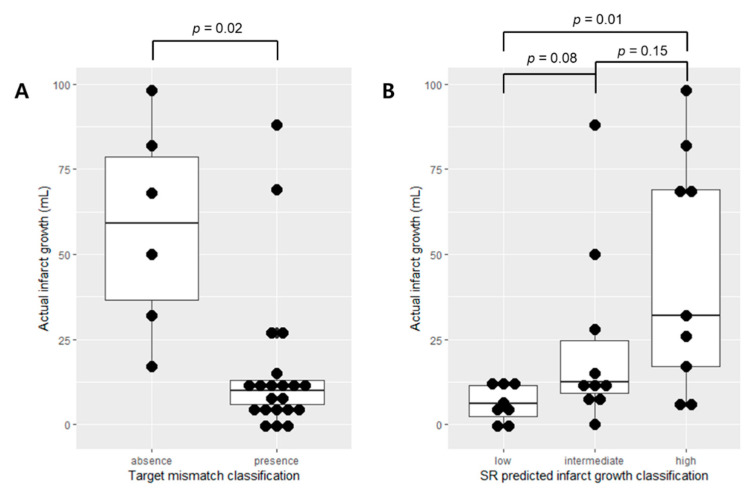
Infarct growth distribution in the external validation SR patients (*n* = 27). (**A**) Target mismatch classification. (**B**) SR-predicted infarct growth classification. In (**A**), the mismatch absence group shows a heterogeneous distribution of actual infarct growth. In (**B**), the high group (SR-predicted infarct growth ≥ 35 mL) shows a heterogeneous distribution of actual infarct growth, and the low group (SR-predicted infarct growth < 15 mL) shows the narrowest distribution of the actual infarct growth.

In external validation SR patients (*n* = 27), there was a statistically significant difference in actual infarct growth volume between the mismatch and non-mismatch groups (*p* = 0.02; Figure 4A). It was also observed that there was a statistically significant difference in actual infarct growth volume between the low and high SR predicted infarct growth volume groups (*p* = 0.01; Figure 4B). *p*-values between the low and intermediate groups and between the intermediate and high groups were 0.08 and 0.15, respectively (Figure 4B).

**Figure 5:**
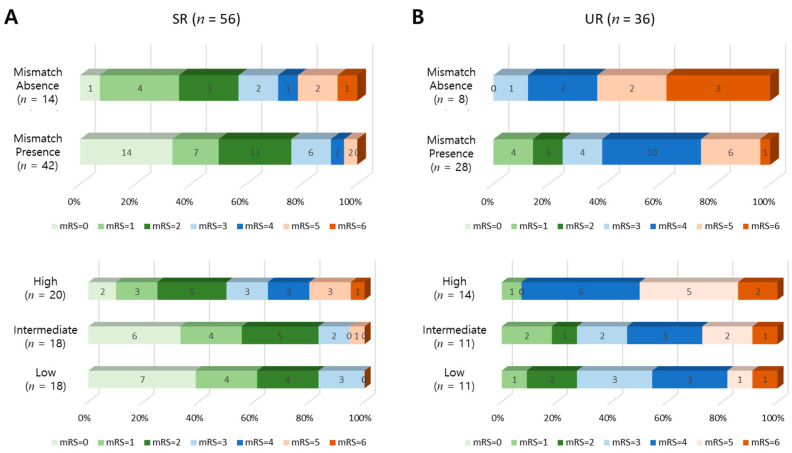
Distribution of modified Rankin Scale (mRS) scores at day-90 by target mismatch (top) and ML-predicted infarct growth (bottom). In both (**A**) SR and (**B**) UR patients, a lower ML-predicted infarct growth was related to a lower mRS score at day 90.

The percentage values of favorable clinical outcomes of the day-90 mRS score ≤ 1 for the mismatch presence and absence groups in overall SR patients were 50% and 36%, respectively (Figure 5A). The percentage values of favorable clinical outcomes were 61%, 56%, and 25%, respectively, for the low, intermediate, and high SR-predicted infarct growth groups (Figure 5A). In UR patients, the percentage values of favorable outcomes for the mismatch presence and absence groups were 14% and 0%, respectively, while they were 9%, 18%, and 7%, respectively, for the low, intermediate, and high UR-predicted infarct growth groups (Figure 5B).

## 4. Discussion

This study suggests that the ML-predicted infarct growth volumes can offer a novel insight into selecting patients and predicting clinical outcome. The method may be a useful alternative to target mismatch, as it provides a direct way to measure infarct distribution and growth. Although the number of the mismatch absence group is small in this study, it indicates that the patient selection based on the ML-predicted infarct growth volume may have the infarct growth prediction performance comparable to target mismatch-based selection. In particular, the low infarct growth group showed a narrow distribution of the actual infarct growth, and this may indicate that once a patient was classified into the low group, a small infarct growth as well as a favorable clinical outcome would be highly likely. It is important to note that traditional PWI–DWI mismatch is evaluated only based on baseline DWI and PWI images without consideration of the final infarct images, while, in the presented approach, the SR and UR infarct volumes are estimated using ML models trained on baseline DWI and PWI images and the final infarct annotated images.

Ischemic penumbra is defined as a hypoperfused area that can regain function with rapid reperfusion [20]. This area is at a risk of infarction without rapid reperfusion, and infarct growth occurs in the absence of reperfusion. In the present study, mean infarct growth in the UR group was higher than that in the SR group, but the degree of infarct growth was highly variable in both UR and SR groups. The large variability of infarct growth within the SR (or UR) group may be attributed to the variable degrees of perfusion defect severities and ischemic damage in the penumbra zone or ischemic core.

Traditional threshold-based lesion volume estimation approaches have limitations, since the appropriate threshold values often depend on MRI sequence parameters, scanner vendors, and software analysis tools. ML takes a different perspective by learning a non-linear function from input/output relationships and has the potential to overcome the limitations of traditional threshold-based volume estimation approaches. Although the evaluation regarded traditional threshold-based methods used for target mismatch assessment as a reference, it is expected that the ML-based method could provide additional information in cases where threshold-based volume estimation is unsatisfactory. Until now, the Diffusion and perfusion imaging Evaluation For Understanding Stroke Evolution (DEFUSE) trial, Echo-Planar Imaging Thrombolytic Evaluation Trial (EPITHET), and other studies have attempted to find the optimal Tmax threshold and mismatch ratio [1,2,21,22]. Similarly, Olivot and colleagues showed that besides mismatch volume, lesion geography and structure also determine infarct growth [23]. Lastly, the rate of infarct growth during the few days after stroke onset varies substantially between patients, and certain patients have slow infarct growth while others have rapid infarct growth [24]. ML-based prediction has the potential to overcome the limitations of traditional approaches of estimating ischemic penumbra.

Current mismatch concepts provide information on the likelihood ratio and number needed to treat for a favorable outcome. In the Highly Effective Reperfusion evaluated in Multiple Endovascular Stroke trials (HERMES), a meta-analysis of individual patient data from five randomized trials of EVT, the odds ratio was 2.49 and the number needed to treat with EVT to reduce disability by at least one level of the mRS for one patient was 2.6 [25]. On the contrary, our ML-based prediction method can produce the visualization of the degree of DWI lesion growth, which can be shown to patients or their guardians, prior to revascularization therapy. ML predictions using our custom tool via post processing typically took approximately 8 min. Using the current strict recanalization criteria based on the mismatch concept, we can calculate treatment effect sizes, but at the cost of limiting the benefit to a small portion of our stroke patients [26]. With ML, treatment approaches may be more customized, rather than dichotomized into either withholding or offering treatment. In addition, Oppenheim and colleagues showed that in patients without SR, DWI volume > 145 mL within 14 h of onset reliably predicted a malignant MCA infarction [27]. ML-based prediction of infarct volume at the peak time of cytotoxic edema (at 3–4 days after the infarction) may guide early management, such as decompressive hemicraniectomy.

This study has several limitations. First, a larger mean difference of predicted infarct volume was observed for UR than for SR. The inaccuracy may be attributed to the large variability of infarct growth rate, which varies from person to person and is highly unpredictable [28]. It may be worth investigating the division of patient grouping based on the infarct growth rate and develop individual ML models. Second, a custom software tool was used to measure the pre-treatment PWI and DWI lesion volumes based on Tmax and ADC thresholds, for the target mismatch assessment. ML-based methods for lesion volume estimation may be alternative tools for mismatch evaluation [29,30]. Third, only ADC and rTTP were used for the ML model development in the present study. Consideration of other MRI sequences, such as fluid-attenuated inversion recovery (FLAIR), may improve the accuracy of infarct growth prediction [31]. Onset to imaging time can affect the volume of the ischemic penumbra zone, as well as the final infarct volume. Recently, the infarct growth rate estimated from the baseline DWI lesion volume and time of stroke onset was reported to be associated with penumbral salvage and clinical outcomes after EVT reperfusion [28]. In a similar fashion, we are currently investigating the feasibility of an improved prediction of infarct growth with the incorporation of relative FLAIR, which is indicative of a ‘tissue clock’, into the prediction model. Fourth, Lev and colleagues reported the importance of ‘location-weighted’ scoring over simple volumetric data of penumbra areas [32,33]. For instance, in patients with similar infarct volumes, different severities of neurologic deficits can be observed, depending on the clinical features and lesion site. Hence, location-weighted ML-based prediction of functional outcome is worthy of investigation. Fifth, clinical variables were not used in this study. Previous studies demonstrated the effectiveness of clinical variables in the prediction of infarct growth [34] and clinical outcome [35], while our study was solely based on image features. The inclusion of clinical variables may help improve the outcome prediction. Finally, this was a single-center study performed on a 3T scanner, with only a small number of patients available for analysis. A larger prospective study is necessary to evaluate the benefit of the ML-based method.

## 5. Conclusions

ML-based prediction of tissue fate was demonstrated using two ML models trained on data from patients with and without recanalization. The presented ML-based method provides the estimations of voxel-wise infarct distributions and final infarct volumes in cases of SR and UR. The classification of patients in terms of ML-predicted infarct growth offers novel and alternative ways to predict infarct growth and functional outcome, when compared with the traditional target mismatch classification. As ML is data-driven and allows sophisticated feature engineering, the ML prediction is expected to improve as more data are collected and careful ML modeling is made based on multi-modal MRI. The advancement of technology will implicate more accurate predictions of the final infarct distribution, potentially providing clinicians with precise information for the guidance of treatment selection and the prediction of clinical outcome.

## Figures and Tables

**Table 1:** Patient characteristics for machine learning (ML) model development and external validation.

	SR (mTICI 2b−3)			UR (mTICI 0–2a)		
	Development	External Validation	*p*-value	Development	External Validation	*p*-value
Number of patients	29	27	-	24	12	-
Age (years), mean ± SD	63 ± 13	67 ± 11	0.17	64 ± 14	67 ± 18	0.62
Male sex, *n* (%)	19 (66)	15 (56)	0.46	17 (71)	7 (58)	0.49
NIHSS score at baseline *	15 (11–18)	16 (10–20)	0.84	13 (9–18)	18 (15–19)	0.03
Onset to MRI time (m) *	131 (99–173)	97 (70–140)	0.20	187 (113–225)	138 (124–216)	0.75
DWI lesion vol (mL), initial *	15 (6–33)	11 (5–28)	0.88	9 (5–18)	15 (6–38)	0.58
PWI lesion vol (mL), initial *	93 (59–133)	104 (72–146)	0.73	83 (53–143)	96 (81–125)	0.93
DWI lesion vol (mL), day-7 *	17 (8–57)	21 (14–79)	0.20	61 (32–118)	124 (84–173)	0.12
Mode of treatment						
No, *n* (%)	0 (0)	0 (0)	-	4 (17)	0 (0)	-
IV tPA only, *n* (%)	0 (0)	0 (0)	-	1 (4)	3 (25)	-
EVT ± IV tPA, *n* (%)	29 (100)	27 (100)	-	19 (79)	9 (75)	-

* Median (IQR), Abbreviations: SR, successful recanalization; UR, unsuccessful recanalization; mTICI, modified treatment in cerebral infarction; SD, standard deviation; NIHSS, National Institutes of Health Stroke Scale; IQR, interquartile range; MRI, magnetic resonance imaging; DWI, diffusion-weighted imaging; PWI, perfusion-weighted imaging; IV, intravenous; tPA, tissue plasminogen activator; EVT, endovascular treatment.

## Appendix A

### Comparison of Tmax > 6 s Volume and rTTP > 4.5 s Volume

A custom MATLAB (Mathworks, Natick, MA, USA) module was developed to calculate Tmax from PWI time series image data and compare it with rTTP. An axial slice with middle cerebral arteries was selected, and an arterial input function (AIF) was automatically calculated by the cluster analysis proposed by Mouridsen et al. [36]. Standard singular value decomposition (SVD) [37] was used to estimate Tmax, defined to be the delay of the peak of the residue function. Figure A1-A compares Tmax > 6 s volume with rTTP > 4.5 s volume. Pearson correlation coefficient between Tmax > 6 s and rTTP > 4.5 s volumes was 0.84 (95% CI = 0.75–0.89, *p*-value < 0.0001). The mean difference (95% limits of agreement from Bland-Altman analysis) was −2.4 mL (−66.3 mL, 61.5 mL). Figure A1-B compares Tmax > 10 s volume with rTTP > 9.5 s volume. Pearson correlation coefficient between Tmax > 10 s and rTTP > 9.5 s volumes was 0.81 (95% CI = 0.72–0.87, *p*-value < 0.0001). The mean difference (95% limits of agreement from Bland–Altman analysis) was 7.5 mL (−42.9 mL, 57.9 mL).
